# Treatment of schizophrenia with antipsychotics in Norwegian emergency wards, a cross-sectional national study

**DOI:** 10.1186/1471-244X-9-24

**Published:** 2009-05-16

**Authors:** Rune A Kroken, Erik Johnsen, Torleif Ruud, Tore Wentzel-Larsen, Hugo A Jørgensen

**Affiliations:** 1 Division of Psychiatry, Haukeland University Hospital, PO Box 23, N-5812, Bergen, Norway; 2 Department of Clinical Medicine, Section Psychiatry, University of Bergen, N-5020 Bergen, Norway; 3 Division of Mental Health Services, Department of Research and Development, Akershus University Hospital, 1478 Lørenskog, Norway; 4 SINTEF Health Research, 0314 Oslo, Norway; 5 Centre for Clinical Research, Haukeland University Hospital, N-5021 Bergen, Norway

## Abstract

**Background:**

Surveys on prescription patterns for antipsychotics in the Scandinavian public health system are scarce despite the prevalent use of these drugs. The clinical differences between antipsychotic drugs are mainly in the areas of safety and tolerability, and international guidelines for the treatment of schizophrenia offer rational strategies to minimize the burden of side effects related to antipsychotic treatment. The implementation of treatment guidelines in clinical practice have proven difficult to achieve, as reflected by major variations in the prescription patterns of antipsychotics between different comparable regions and countries. The objective of this study was to evaluate the practice of treatment of schizophrenic patients with antipsychotics at discharge from acute inpatient settings at a national level.

**Methods:**

Data from 486 discharges of patients from emergency inpatient treatment of schizophrenia were collected during a three-month period in 2005; the data were collected in a large national study that covered 75% of Norwegian hospitals receiving inpatients for acute treatment. Antipsychotic treatment, demographic variables, scores from the Global Assessment of Functioning and Health of the Nation Outcome Scales and information about comorbid conditions and prior treatment were analyzed to seek predictors for nonadherence to guidelines.

**Results:**

In 7.6% of the discharges no antipsychotic treatment was given; of the remaining discharges, 35.6% were prescribed antipsychotic polypharmacy and 41.9% were prescribed at least one first-generation antipsychotic (FGA). The mean chlorpromazine equivalent dose was 450 (SD 347, range 25–2800). In the multivariate regression analyses, younger age, previous inpatient treatment in the previous 12 months before index hospitalization, and a comorbid diagnosis of personality disorder or mental retardation predicted antipsychotic polypharmacy, while previous inpatient treatment in the previous 12 months also predicted prescription of at least one FGA.

**Conclusion:**

Our national survey of antipsychotic treatment at discharge from emergency inpatient treatment revealed antipsychotic drug regimens that are to some degree at odds with current guidelines, with increased risk of side effects. Patients with high relapse rates, comorbid conditions, and previous inpatient treatment are especially prone to be prescribed antipsychotic drug regimens not supported by international guidelines.

## Background

The clinical differences between antipsychotic drugs are mainly in the areas of safety and tolerability. International guidelines for the treatment of schizophrenia [1-4] offer rational strategies to minimize the burden of side effects related to antipsychotic treatment. These recommendations may be considered according to three dimensions: first-versus second-generation antipsychotics; antipsychotic mono-versus polypharmacy; and optimal dosing of antipsychotics. The second-generation antipsychotics (SGAs) as a group are less associated with extrapyramidal symptoms (EPS) and hyperprolactinemia compared with the older first-generation drugs (FGAs), and are recommended by most guidelines as first-line therapy for schizophrenia [3]. Antipsychotic monotherapy is generally recommended, and most guidelines converge on a recommendation that doses should be between 300 and 1000 chlorpromazine equivalents [5] in order to achieve the important goal of maximum reduction of positive symptoms, while avoiding EPS and use of anticholinergic medication [6].

The implementation of treatment guidelines in clinical practice has proven difficult to achieve [7], reflected by major variations in the prescription patterns of antipsychotics between different comparable regions and countries [8-10]. Prescribing a drug regime with poorer tolerability may ultimately increase the risk of relapse, as an association between increasing side effects and poorer drug adherence has been shown [11]. The goal of avoiding relapses and hospital admissions for patients with schizophrenia is also of major importance from a cost-effectiveness perspective as readmissions represent one of the largest contributors to total treatment costs [12]. Surveys of prescription patterns for antipsychotics in the Scandinavian public health system are scarce despite the prevalent use of these drugs. Studies addressing issues related to optimal antipsychotic treatment, as well as detection of predictors of noncompliance to guidelines, are important. The psychiatric emergency wards often set the stage for the treatment of schizophrenia and offer an important opportunity to investigate the prescription patterns of antipsychotics.

The aims of this study were to evaluate the Norwegian practice of antipsychotic treatment of patients with schizophrenia at discharge from acute inpatient settings, compared with the international recommended guidelines of monotherapy, choice of antipsychotics, and dosing. In addition, we wanted to study whether demographic or clinical factors at admission predicted noncompliance to guideline recommendations.

## Methods

The data were collected in the Norwegian Multicenter Study in Acute Psychiatry (MAP) [13] conducted by the Network for Evaluation of Acute Psychiatry, coordinated by SINTEF Health Research and financed in part by the Norwegian Directorate of Health and Social Affairs. The study was approved by the Regional Ethical Committee, the Norwegian Directorate of Health and Social Affairs, and the Norwegian Data Inspectorate. The Norwegian psychiatric inpatient system receives mostly acutely admitted patients to emergency wards at psychiatric hospitals or departments at general hospitals. The majority of all admitted patients are discharged from these wards to outpatient treatment in the psychiatric specialist system or to treatment by general practitioners. Many of the patients with schizophrenia or major affective disorders are transferred to other specialized hospital wards or community mental health centers for further inpatient treatment. MAP is a cross-sectional observational study that included 19 hospitals with 37 inpatient wards receiving acutely admitted patients. The emergency wards were distributed in the five health regions of Norway, and the participating hospitals were university hospitals and regional and local psychiatric hospitals. A total of 3506 consecutive hospital admissions were recorded during three months of 2005. The 19 participating hospitals comprised approximately 75% of the Norwegian hospitals receiving patients for acute psychiatric inpatient treatment. From this material we have selected admissions with a diagnosis of schizophrenia at discharge (some patients had more than one admission and discharge in the study period). Data were recorded at admission and discharge.

The data collected by MAP at admission and discharge from emergency wards included demography, information about treatment history, medication, and clinical measures including the Global Assessment of Function-Split Version (S-GAF) [14,15] and Health of the Nation Outcome Scales (HoNOS) [16]. The rating on the scales was performed by the resident who assessed the patient at admission to the hospital, and training in HoNOS scoring was conducted at all participating hospitals. HoNOS item 1 (overactive, aggressive, disruptive, or agitated behavior) and HoNOS item 6 (hallucinations and delusions) were dichotomized to 0 (scores of 0, 1, or 2) or 1 (scores of 3 or 4). Clinical diagnoses according to the ICD 10 [17] were obtained from the medical records. Classification of antipsychotics in FGAs or SGAs and the computation of chlorpromazine-equivalent doses (CPZ) were done according to the literature and listed in Table 1[18-24]. The Defined Daily Dose (DDD) is obtained from the WHO Collaborating Centre for Drug Statistics Methodology and is assumed to be the average maintenance dose per day for a drug used for its main indication in adults[22]. To focus on prescriptions intended to treat psychotic symptoms, we excluded prescriptions of low-potency FGAs in doses below 100 CPZ from the analyses of prediction of polypharmacy and prediction of prescription of at least one FGA at discharge.

**Table 1:** Antipsychotic equivalent doses and Defined Daily Doses of common antipsychotics.

Drug	Potency Ratio	Antipsychotic equivalent doses.	DDD^5 ^(mg)	Adm-route
**FGA**				
**Low-potency**				
Chlorpromazine (Largactil^®^)	1.0	300.0	300.0	O
Klorprotixene (Truxal ^®^)	2.0	150.0^2^	300.0	O
Levomepromazine (Nozinan^®^)	1.0	300.0^5^	300.0	O
Thioridazine (Melleril^®^)	1.0	300.0^1^	300.0	O
**Medium potency**				
Dixyrazine (Esucos^®^)	6.0	50.0^5^	50.0	O
Perphenazine (Trilafon^®^)	12.5	24.0^1^	30.0	O
Perphenazine decanoate (Trilafon depot^®^)	53.0	5.7^5^	7.0	P
Prochlorperazine (Stemetil^®^)	6.7	45.0^1^	100.0	O
Zuclopenthixol (Cisordinol^®^)	4.0	75.0^1^	30.0	O
Zuclopenthixol decanoate (Cisordinol Depot^®^)	7.0	42.0^1^	15.0	P
**High-potency**				
Flupenthixol (Fluanxol^®^)	50.0	6.0^1^	6.0	O
Flupenthixol decanoate (Fluanxol depot^®^)	70.0	4.2^1 ^^4^	4.0	P
Haloperidol (Haldol^®^)	33.0	9.0^1^	8.0	O
Haloperidol decanoate (Haldol depot^®^)	50.0	6.0^1 ^^4^	3.3	P
**SGA**				
Amisulpride (Solian^®^)	1.0	300.0^1^	400.0	O
Aripiprazole (Abilify^®^)	13.3	22.5^3^	15.0	O
Clozapine (Leponex^®^, Clozapine^®^)	1.0	300.0^1^	300.0	O
Olanzapine (Zyprexa^®^)	20.0	15.0^3^	10.0	O
Quetiapine (Seroquel^®^)	1.3	225.0^3^	400.0	O
Risperidone (Risperdal^®^)	66.0	4.5^1^	5.0	O
Risperidone long-acting injection (Risperdal Consta^®^)	100.0	3.0^6^	1.8	P
Sertindole (Serdolect^®^)	18.8	16.0^5^	16.0	O
Ziprazidone (Zeldox^®^)	1.6	180.0^3^	80.0	O

^1^Bazire, S. (2005) Psychotropic Drug Directory 2005. [18]

^2^Davis, J. M. (1974) Dose equivalence of the antipsychotic drugs. J. Psychiatr. Res., 11, 65–69[19]

^3^Woods, S. W. (2003) Chlorpromazine equivalent doses for the newer atypical antipsychotics. J. Clin. Psychiatry, 64, 663–667. [20]

^4^Kane, J. M., Aguglia, E., Altamura, A. C., et al (1998) Guidelines for depot antipsychotic treatment in schizophrenia. European Neuropsychopharmacology Consensus Conference in Siena, Italy. Eur. Neuropsychopharmacol., 8, 55–66. [21]

^5^WHO Collaborating Centre for Drug Statistics Methodology (2005) ATC Index with DDDs. [22]

^6 ^Bai YM et al, Equivalent Switching Dose From Oral Risperidone to Risperidone Long-Acting Injection: A 48-Week Randomized, Prospective, Single-Blind Pharmacokinetic Study. J Clin Psychiatry 2007;68:1218–1225. [24].

^7^Administration Route: O: oral, P: peroral.

The chlorpromazine equivalent doses of levomepromazine, dixyrazine, sertindol and perphenazine decanoate are not adequately defined in the literature, and chlorpromazine equivalent dose is calculated from the DDD.

### Statistical analysis

All regression analyses used methods for clustered observations (GEE) because some patients had more than one hospitalization. For each drug, the change in prescriptions between admission and discharge was tested by logistic regression. The *p *values were adjusted for multiple comparisons with the Benjamini-Hochberg method.

Univariate and multivariate logistic regression analyses with polypharmacy at discharge and use of at least one FGA at discharge as dependent variables (after exclusion of prescriptions of low-potency FGAs in doses below 100 CPZ) were conducted with the following independent variables: age, sex, GAF severity of symptoms at admission, HoNOS items 1 and 6 (dichotomized) at admission, inpatient and outpatient treatment during the immediate 12 months before admission (dichotomized), having a remitting/chronic or first-episode condition, and having a comorbid diagnosis of substance abuse, personality disorder, or mental retardation. However, in the logistic regression with use of at least one FGA at discharge as the dependent variable, recurrent illness and a comorbid diagnosis of mental retardation had to be excluded from the analysis for stability reasons. As sensitivity analyses, the analyses were repeated, including low-potency FGAs. Results are presented as odds ratios with corresponding 95% confidence intervals and *p *values.

The relationship between dosage and the independent variables above was analyzed using linear regression. Results were presented with *p *values, and effects were reported with unstandardized regression coefficients and 95% confidence intervals. The relationships between type (university vs. other) and region of the hospitals, and use of polypharmacy and at least one FGA were analyzed by logistic regression. SPSS software, version 15 (SPSS, Chicago, IL, USA) and R (The R Foundation for Statistical Computing, Vienna, Austria) were used for the statistical analyses.

## Results

A total of 486 discharges of patients with a diagnosis of schizophrenia were identified and selected from the MAP database. The discharges were distributed among 412 patients where 352 patients had one hospitalization, 49 patients had two hospitalizations, eight patients had three hospitalizations, and three patients had four hospitalizations in the acute inpatient units during the three-month study period. The background variables are given in Table 2.

**Table 2:** Background variables of patients with schizophrenia at discharge from acute wards (n = 486).

Sex (M/F)	297/189 (61.1%/38.9%)
Age	40.8 ± 13.5 (range 15-82)
Ethnic Norwegian	430 (88.5%)
First-episode illness	17 (3.5%)
S-GAF: severity of symptoms at admission	31.7 ± 10.2 (range 10–75)
S-GAF: severity of functioning at admission	33.5 ± 10.3 (range 10–75)
Aggression at admission (HoNOS 1)	86 (17.7%)
Delusions/hallucinations at admission (HoNOS 6)	291 (59.9%)
Outpatient treatment in the previous 12 months	258 (53.1%)
Inpatient treatment in the previous 12 months	341 (70.2%)
Comorbid diagnosis of drug use/addiction	55 (11.3%)
Comorbid personality disorder	8 (1.6%)
Comorbid diagnosis of mental retardation	9 (1.9%)
Transferred to further in-patient treatment at discharge	286 (59.5%)

Olanzapine was the single most frequently prescribed drug both at admission (25.1%) and at discharge (29.0%), followed by risperidone, which was prescribed in 19.1% of the discharges. There was a significant increase in the prescription of olanzapine (*p *= 0.049) and zuclopenthixol (*p *= 0.049) from admission to discharge (see Table 3). Depot antipsychotics were prescribed in 35.6%, and clozapine was prescribed in 8.0% of the discharges.

**Table 3:** Prescription of antipsychotic drugs at admission and discharge, p-values of differences and chlorpromazine equivalent doses at discharge (n = 486).

Antipsychotic drug	Admission N(%)	Discharge N(%)	Adjusted P-value for the difference*	Chlorpromazine equivalent dose at discharge mean (SD) range
**FGA**				
**Low-potency**				
Levomepromazine (Nozinan^®^)	31(6.4)	39(8.2)	0.248	87 (99) 10–400
Chlorpromazine (Largactil^®^)	29(6.0)	28(5.8)	0.793	171 (104) 4–400
Klorprotixene (Truxal ^®^)	13(2.7)	18(3.7)	0.501	250 (145) 111–667
Thioridazine (Melleril^®^)	1(0.2)	1(0.2)		75
**Medium potency**				
Perphenazine (Trilafon^®^)	24(4.9)	17(3.5)	0.248	187 (121) 50–400
Perphenazine decanoate (Trilafon depot^®^)	37(7.6)	49(10.1)	**0.049**	488 (243) 203–1624
Perphenazine total number patients	60(12.4)	65(13.4)	0.537	
Zuclopentixol (Cisordinol^®^)	15(3.1)	21(4.3)	0.501	58 (27) 28–128
Zuclopenthixol decanoate (Cisordinol Depot^®^)	39(8.0)	49(10.1)	0.247	115 (61) 34–357
Zuclopenthixol total number patients	51(10.5)	68(14.0)	**0.049**	
Dixyrazine (Esucos^®^)	4(0.8)	6(1.2)	0.536	300 (300-300)
Prochlorperazine (Stemetil^®^)	1(0.2)	1(0.2)		1000
**High-potency**				
Haloperidol (Haldol^®^)	8(1.7)	10(2.0)	0.613	144 (85) 33–267
Haloperidol decanoate (Haldol depot^®^)	6(1.2)	7(1.4)	0.524	400 (164) 179–714
Haloperidol total number patients	14(2.9)	15(3.1)	0.793	
Flupenthixol (Fluanxol^®^)	2(0.4)	3(0.6)	0.537	266 (85) 33–267
Flupenthixol decanoate (Fluanxol depot^®^)	5(1.0)	6(1.2)	0.793	247 (129) 153–429
Flupentixol total number patients	7(1.4)	9(1.9)	0.613	
**SGA**				
Olanzapine (Zyprexa^®^)	122 (25.1)	141 (29.0)	**0.049**	350 (137) 100–800
Risperidone (Risperdal^®^)	39(8.0)	43(8.9)	0.613	234 (107) 67–467
Risperidone long-acting injection (Risperdal Consta^®^)	57(11.7)	62(12.8)	0.565	311 (94) 179–536
Risperidon total number patients	91 (18.7)	93 (19.1)	0.793	
Quetiapine (Seroquel^®^)	42(8.6)	46(9.5)	0.613	690 (533) 33–2800
Clozapine (Leponex^®^, Clozapine^®^)	37(7.6)	39(8.0)	0.524	
Aripiprazole (Abilify^®^)	29(6.0)	30(6.2)	0.793	285 (94) 133–400
Ziprazidone (Zeldox^®^)	23(4.7)	24(4.9)	0.793	173 (90) 33–266
Amisulpride (Solian^®^)	11(2.3)	14(2.9)	0.537	600 (353) 100–1200
Sertindole (Serdolect^®^)	0(0)	1(0.2)		missing
Total dose				450 (347) 25–2800

* Logistic regression for repeated observations (GEE). Exchangeable working correlations were used except (for stability reasons) for haloperidol depot and clozapine. For prochlorperazine and sertindol no tests were performed (at most one occasion of use at admission resp. discharge). The results were adjusted for multiple comparisions with Benjamini-Hochberg's method.

In 7.6% of the cases, no antipsychotic medication was prescribed at discharge. In the remaining 449 discharges, antipsychotic polypharmacy was used in 160 cases (35.6%) and one or more FGA in 204 (41.9%) cases. In a subanalysis with exclusion of FGA in doses below 100 CPZ, antipsychotic polypharmacy was prescribed in 112 cases (24.9%) and at least one FGA in 177 cases (36.4%).

Of the total of 449 discharges with antipsychotic treatment, monotherapy was prescribed in 289 (64.4%) of the cases; of these, 203 (45.2%) were treated with SGAs. Among the 160 (35.6%) cases with polypharmacy, 29 (6.5%) were treated with combinations of two or more FGAs; two or more SGAs were prescribed in 42 (9.4%) cases; and in 89 cases (19.8%), a combination including both FGA and SGA was prescribed. In 56 (12.3%) periods for patients treated with antipsychotic polypharmacy, one or more antipsychotics were started during the hospitalization, combined with one or more antipsychotics unchanged from admission to discharge. In 20 (4.5%) cases, patients received clozapine in combination with another SGA.

There were 15 cases with first-episode patients. Of these, 14 were treated with SGA monotherapy and one had a combination of SGA with an FGA (25 mg levomepromazine).

In seven of the nine discharges with a comorbid diagnosis of mental retardation, an FGA was prescribed.

In the univariate analyses, predictors for antipsychotic polypharmacy (excluding low-potency antipsychotics in doses below 100 CPZ) were found to be younger age, lower GAF-S score, recurrent illness, having had inpatient treatment in the previous 12 months, a comorbid personality disorder, and mental retardation. In the multivariate analyses, younger age, inpatient treatment during the previous 12 months, and a comorbid diagnosis of a personality disorder or mental retardation predicted antipsychotic polypharmacy (See Table 4). In sensitivity analysis including low-potency FGAs, only personality disorder was significant (OR 7.16, *p *= 0.035). Female sex and inpatient treatment in the previous 12 months were found to be predictors for the prescription of at least one FGA (excluding low-potency antipsychotics in doses below 100 CPZ) at discharge in the univariate analyses. In the multivariate analyses, only inpatient treatment in the previous 12 months was found to predict FGA prescription. In sensitivity analysis including low-potency FGAs, personality disorders had to be excluded for stability reasons. As in the original model, in-patient treatment during the previous 12 months was still the only significant predictor (OR 1.93, *p *= 0.014).

**Table 4:** Prediction of antipsychotic polypharmacy and prescription of at least one FGA at discharge.

		Prediction of FGA 95,0% C.I. for OR.				Prediction of polypharmacy 95,0% C.I. for OR.		
						
Variables	OR (Odds Ratio)	Lower	Upper	P-value	OR (Odds Ratio)	Lower	Upper	P-value
Age.	1.011	0.993	1.029	0.228	0.979	0.959	0.999	**0.036**
Female sex.	1.435	0.864	2.384	0.163	0.810	0.459	1.431	0.469
S-GAF: severity of symptoms at admission.	0.992	0.971	1.013	0.449	0.984	0.960	1.007	0.181
Aggression at admission (HoNOS-1).	1.292	0.715	2.335	0.397	1.056	0.534	2.085	0.876
Delusions/hallucinations at admission (HoN0S-6).	0.754	0.483	1.178	0.215	0.897	0.522	1.540	0.693
Recurrent illness.	*	*	*	*	4.731	0.572	39.168	0.150
Outpatient treatment in the previous 12 months.	0.769	0.466	1.267	0.302	0.678	0.396	1.162	0.157
In-patient treatment in the previous 12 months.	2.220	1.259	3.915	**0.006**	2.396	1.087	5.282	**0.030**
Comorbid diagnosis of drug misuse/addiction.	0.624	0.264	1.470	0.281	1.342	0.520	3.460	0.543
Comorbid diagnosis of personality disorder.	1.793	0.481	6.691	0.385	5.005	1.211	20.676	**0.026**
Comorbid diagnosis of mental retardation.	*	*	*	*	13.968	1.341	145.485	**0.027**

Results from multivariate logistic regressions.

*Deleted from the model for model stability reasons.

Mean chlorpromazine equivalent dose at discharge was 450 CPZ (SD 347, range 25–2800) (see Table 3). In the linear regression with dosage as the dependent variable, lower age, delusions or hallucinations at admission, recurrent illness, and inpatient treatment during the previous year were found to predict higher dosages.

There were significant regional differences in the use of FGAs (*p *= 0.012), with more prevalent prescription of FGAs in the southern (OR 3.21) and northern (OR 1.88) regions compared with the eastern region. No similar relationship was observed for the use of intended polypharmacy (*p *= 0.412), and there were no significant differences between departments in university hospitals and other hospitals.

## Discussion

The present study revealed a total rate of 35.6% of polypharmacy with antipsychotics and a rate of 41.9% first-generation antipsychotics at discharge. The antipsychotic drug doses were within the recommended range in most cases. An argument for not classifying low-potency FGAs (levomepromazine, chlorpromazine, chlorprothixene, and thioridazine) at low doses as antipsychotics exists if the definition applies only to drugs prescribed for their antipsychotic properties. It is not uncommon to use low-potency FGAs as hypnosedatives, and in this regard, they should not be classified as antipsychotics in the strict sense of the term. In practice, however, such a definition may prove difficult to validate retrospectively, as one would need to know the intent of each prescribing clinician to decide whether the drugs were prescribed for psychosis *per se*. When low-potency FGAs in doses below 100 CPZ were excluded from the analyses in our sample, the rate of polypharmacy was reduced to 24.9%, and the proportion of periods prescribed at least one FGA was 36.4%. Regarding antipsychotic polypharmacy, our findings are comparable to those of other studies from Norway [8], Denmark [25], Italy [26], and North America [27], in which rates have ranged from 27% to 48%. A study from Innsbruck reported only 12% antipsychotic polypharmacy at discharge; in this study low-potency FGAs were excluded from the analyses. However, an increasing trend of polypharmacy in recent years was also identified in that study [28]. One previous study indicates that the level of polypharmacy decreases when patients are followed-up after discharge, and that part of polypharmacy can be an intermediate situation [29]. The risk is, however, of long-term continuation of polypharmacy after discharge from hospitalization because of sparse follow-up [30]. Nearly 5% of discharges have combinations of an SGA with clozapine, which could be in line with guideline recommendations in situations where clozapine monotherapy has been tried without success. The 2006 Update of the Texas Medication Algorithm for Schizophrenia recommends a trial of combination with another antipsychotic when there is partial or no response to clozapine, although the results from randomized controlled trials in this field are inconsistent [1]. Our data did not give answers to the questions about treatment resistance; however, it is not unlikely that the finding of a rate of polypharmacy of 35.6% (24.9% when low-potency FGAs in doses below 100 CPZ are excluded) reflects a high rate of treatment resistance. This is also supported by the finding that 70.2% of patients had inpatient treatment in the previous 12 months. Bearing this in mind, the finding that only 8.0% of patients were prescribed clozapine can indicate a prescription rate of clozapine that was too low. The finding of a rate of 6.5% discharges with a combination of two or more FGAs, and 19.8% with a combination of an FGA and an SGA are indications of guideline noncompliance.

More than 40% of cases were prescribed at least one FGA at discharge in our study (36.4% when FGAs in doses below 100 CPZ were excluded). This is indeed a very high rate compared with findings from the USA [31], while comparable to previous findings from European studies [8,26]. In the recent World Psychiatric Association Pharmacopsychiatry Section statement on comparative effectiveness of antipsychotics in the treatment of schizophrenia [5], the goal to achieve a maximum reduction of positive symptoms while avoiding EPS or the use of anticholinergic medication is emphasized, and while the possibility of achieving this with FGA treatment is noted, the probability of reaching this goal is more likely with SGA treatment. A newly published review finds FGAs to be associated with more extrapyramidal side effects compared with SGAs [32], which is also the case for tardive dyskinesias [33]. The high rate of FGA prescription in our study could indicate suboptimal practice, leaving the patients at higher risk of EPS than necessary. In addition to this, the two most prescribed SGAs in our sample at discharge were olanzapine (29.0%) and risperidone (19.1%). These agents have both gained attention because of their side-effect profiles that include weight gain, dyslipidemia, and diabetogenic effect for olanzapine, and hyperprolactinemia and EPS for risperidone. The high prescription rates of FGA and the SGAs olanzapine and risperidone may indicate that side-effect considerations are not prioritized in the planning of antipsychotic treatment. A further indication is the significant rise in the prescription of the FGA zuclopenthixol between admission and discharge, as zuclopenthixol treatment has a high risk of extrapyramidal side effects.

Younger age, inpatient treatment in the previous 12 months, as well as comorbid disorders predicted polypharmacy at discharge, while inpatient treatment during the previous 12 months predicted the prescription of FGA at discharge. Although a comorbid diagnosis of mental retardation had to be excluded from the regression with FGA as the dependent variable, there is a strong covariation as we found that an FGA was prescribed in seven out of nine discharges with a comorbid mental retardation. Bearing in mind that our data do not provide information about treatment resistance, a reasonable interpretation of these findings may be that psychiatrists working in acute inpatient units combine two or more antipsychotics when the mental illness is complex, chronic, or remitting, while higher symptom burden measured by GAF or HoNOS at admission does not in itself predict either polypharmacy or prescription of FGA. This is in line with findings in a large study of Veteran Administration patients followed for one year [34]. The fact that younger age predicted polypharmacy is difficult to explain given treatment recommendation of lower doses for first-episode patients. One possible explanation may be that schizophrenia can be associated with more florid symptoms in younger patients.

The clinical significance of the predictive effects of comorbidity with mental retardation is questionable, at least in our data, as the prevalence of this disorder is low. However, the finding of a very high prevalence of both polypharmacy and the use of FGAs in this group is worth noting.

The recent statement from the World Psychiatric Association Pharmacopsychiatry Section [6] also emphasizes dosing as a key variable in optimizing effectiveness of antipsychotic treatment. Although a comprehensive survey of schizophrenia guidelines shows a great variation in methodological quality, there was a remarkable convergence in the recommendation of antipsychotic doses between 300 and 1000 CPZ [5]. However, the authors find in the content analysis of the guidelines that a few reference guidelines seem to have been used as primers for others, which may have caused the convergence. The newest guidelines in the survey recommended lower doses, and a possible danger of higher doses is highlighted in a current study of sudden cardiac death among a large cohort of users of antipsychotics [35]. An overall increased rate of sudden cardiac death for users of antipsychotics versus nonusers was identified, and the risk increased with increasing dose. The dosages of antipsychotic treatment found in the present study were largely in line with present guidelines; however, a substantial portion of discharged patients was treated with doses below 300 CPZ. The interindividual variations in metabolism and bioavailability of antipsychotics is great, and doses below 300 CPZ can be effective, especially in first-episode patients [36]. Other measures for comparing antipsychotic equivalence exist, including Defined Daily Doses (DDD) [37], which may give somewhat different results [18]. Each method has its flaws, however, and there is, in the opinion of the authors, no convincingly better approach to compare doses in different antipsychotic drug regimens.

Regarding prescription patterns between different hospital sites, regional differences were found. However, because of a lack of power, it was not possible to control this finding in a multivariate analysis. The existence of idiosyncratic and local drug practices is found in other studies [38].

Because data were obtained from 19 different departments with 27 different wards, there can be variations in the way HoNOS and GAF were scored, although efforts have been made to minimize this variation. The use of clinical diagnoses at discharge has its limitations, as in all studies based on clinical diagnostic work. The evaluation at admission of seriously disturbed psychotic patients can be difficult and can give incomplete information about prior treatment, and although we tried to collect information from other sources, it is possible that some information is missing. Another limitation in the present study is that a large portion (59.5%) of the patients in our study was transferred to other inpatient treatment when discharged from the acute ward, and thus the prescription at the final discharge from inpatient treatment can differ from our results. In 12% of the cases, a new prescription was added to an antipsychotic drug that was left unchanged during the hospitalization, and some of the cases of antipsychotic polypharmacy may represent a crossover period.

## Conclusion

We conclude that our national survey of antipsychotic treatment from acute inpatient treatment reveal drug regimens that are to some degree at odds with current guidelines, with increased risk of side effects. Patients with a high degree of recidivism, comorbid conditions, and previous inpatient treatment are especially prone to receive deviating antipsychotic drug regimens, which has also been found in other studies [39]. That some aspects of the antipsychotic treatment were in total accordance with guideline recommendations, e.g., the prescription of monotherapy SGA to first-episode patients, could be an indication that the guidelines are well known to the psychiatrists. The reason for nonadherence to guidelines could be vague or missing guideline recommendations for the treatment of the most complex schizophrenic conditions, as underlined by Leucht [7]. This should be a target for future studies.

## Competing interests

The authors declare that they have no competing interests.

## Authors' contributions

RK and TR took part in the data collection, RK drafted the manuscript. RK and TWL performed the statistical analyses, HAJ and EJ helped to draft the manuscript. All authors read and approved the final manuscript.

## Pre-publication history

The pre-publication history for this paper can be accessed here:



## References

[B1] Moore TA, Buchanan RW, Buckley PF, Chiles JA, Conley RR, Crismon ML, Essock SM, Finnerty M, Marder SR, Miller DD, McEvoy JP, Robinson DG, Schooler NR, Shon SP, Stroup TS, Miller AL (2007). The Texas Medication Algorithm Project antipsychotic algorithm for schizophrenia: 2006 update. J Clin Psychiatry.

[B2] Lehman AF, Kreyenbuhl J, Buchanan RW, Dickerson FB, Dixon LB, Goldberg R, Green-Paden LD, Tenhula WN, Boerescu D, Tek C, Sandson N, Steinwachs DM (2004). The Schizophrenia Patient Outcomes Research Team (PORT): updated treatment recommendations 2003. Schizophr Bull.

[B3] National Institute for Clinical Excellence (2002). Schizophrenia Core interventions in the treatment and management of schizophrenia in primary and secondary care.

[B4] Lehman AF, Lieberman JA, Dixon LB, McGlashan TH, Miller AL, Perkins DO, Kreyenbuhl J (2004). Practice guideline for the treatment of patients with schizophrenia, second edition. Am J Psychiatry.

[B5] Gaebel W, Weinmann S, Sartorius N, Rutz W, McIntyre JS (2005). Schizophrenia practice guidelines: international survey and comparison. Br J Psychiatry.

[B6] Tandon R, Belmaker RH, Gattaz WF, Lopez-Ibor JJ, Okasha A, Singh B, Stein DJ, Olie J-P, Fleischhacker WW, Moeller H-J (2008). World Psychiatric Association Pharmacopsychiatry Section statement on comparative effectiveness of antipsychotics in the treatment of schizophrenia. Schizophr Res.

[B7] Leucht S (2007). Psychiatric treatment guidelines: doctors' non-compliance or insufficient evidence?. Acta Psychiatr Scand.

[B8] Johnsen E, Svingen GF, Jorgensen HA (2004). Practice regarding antipsychotic therapy: a cross-sectional survey in two Norwegian hospitals. Nord J Psychiatry.

[B9] Barbui C, Nose M, Mazzi MA, Thornicroft G, Schene A, Becker T, Bindman J, Leese M, Helm H, Koeter M, Weinmann S, Tansella M (2006). Persistence with polypharmacy and excessive dosing in patients with schizophrenia treated in four European countries. Int Clin Psychopharmacol.

[B10] Bitter I, Chou JC, Ungvari GS, Tang WK, Xiang Z, Iwanami A, Gaszner P (2003). Prescribing for inpatients with schizophrenia: an international multi-center comparative study. Pharmacopsychiatry.

[B11] Lambert M, Conus P, Eide P, Mass R, Karow A, Moritz S, Golks D, Naber D (2004). Impact of present and past antipsychotic side effects on attitude toward typical antipsychotic treatment and adherence. Eur Psychiatry.

[B12] Tunis SL, Faries DE, Nyhuis AW, Kinon BJ, Ascher-Svanum H, Aquila R (2006). Cost-effectiveness of olanzapine as first-line treatment for schizophrenia: results from a randomized, open-label, 1-year trial. Value Health.

[B13] Ruud T, Gråwe RW, Hatling T (2006). Acute Psychiatric Treatment in Norway – results from a multicenter study (In Norwegian).

[B14] Karterud S, Pedersen G, Loevdahl H, Friis S (1998). Global Assessment of Functioning – Split Version (S-GAF): Background and Scoring Manual.

[B15] American Psychiatric Assosiation (1994). Diagnostic and Statistical Manual of Mental Disorders.

[B16] Wing JK, Beevor AS, Curtis RH, Park SB, Hadden S, Burns A (1998). Health of the Nation Outcome Scales (HoNOS). Research and development. Br J Psychiatry.

[B17] World Health Organization (1992). The ICD-10 Classification of Mental and Behavioural Disorders, Clinical Description and Diagnostic Guidelines.

[B18] Bazire S (2005). Psychotropic Drug Directory.

[B19] Davis JM (1974). Dose equivalence of the antipsychotic drugs. J Psychiatr Res.

[B20] Woods SW (2003). Chlorpromazine equivalent doses for the newer atypical antipsychotics. J Clin Psychiatry.

[B21] Kane JM, Aguglia E, Altamura AC, Ayuso Gutierrez JL, Brunello N, Fleischhacker WW, Gaebel W, Gerlach J, Guelfi J-D, Kissling W, Lapierre YD, Lindstrøm E, Mendlewicz J, Racagni G, Carulla LS, Schooler NR (1998). Guidelines for depot antipsychotic treatment in schizophrenia. European Neuropsychopharmacology Consensus Conference in Siena, Italy. Eur Neuropsychopharmacol.

[B22] WHO Collaborating Centre for Drug Statistics Methodology (2008). ATC classification index with DDDs 2008 Oslo, Norway.

[B23] Royal Pharmaceutical Society of Great Britain (2008). British National Formulary 2008.

[B24] Bai YM, Ting CT, Chen JY, Chang WH, Wu B, Hung CH, Lin WK (2007). Equivalent switching dose from oral risperidone to risperidone long-acting injection: a 48-week randomized, prospective, single-blind pharmacokinetic study. J Clin Psychiatry.

[B25] Danish National Board of Health (2006). Use of antipsychotics in patients aged 18–64 years with schizophrenia, mania or bipolar affective disorder (In Danish).

[B26] Ballerini A, Boccalon RM, Boncompagni G, Casacchia M, Margari F, Minervini L, Righi R, Russo F, Salteri A, Frediani S, Rossi A, Scatigna M (2007). Clinical features and therapeutic management of patients admitted to Italian acute hospital psychiatric units: the PERSEO (psychiatric emergency study and epidemiology) survey. Ann Gen Psychiatry.

[B27] Procyshyn RM, Kennedy NB, Tse G, Thompson B (2001). Antipsychotic polypharmacy: a survey of discharge prescriptions from a tertiary care psychiatric institution. Can J Psychiatry.

[B28] Edlinger M, Hausmann A, Kemmler G, Kurz M, Kurzthaler I, Walch T, Walpoth M, Fleischhacker WW (2005). Trends in the pharmacological treatment of patients with schizophrenia over a 12 year observation period. Schizophr Res.

[B29] Kreyenbuhl J, Valenstein M, McCarthy JF, Ganoczy D, Blow FC (2006). Long-term combination antipsychotic treatment in VA patients with schizophrenia. Schizophr Res.

[B30] Weiden PJ, Casey DE (1999). "Polypharmacy:" Combining Antipsychotic Medications in the Treatment of Schizophrenia. Jrnl Prac Psych and Behav Hlth.

[B31] Lieberman JA, Stroup TS, McEvoy JP, Swartz MS, Rosenheck RA, Perkins DO, Keefe RSE, Davis SM, Davis CE, Lebowitz BD, Severe J, Hsiao JK (2005). Effectiveness of antipsychotic drugs in patients with chronic schizophrenia. N Engl J Med.

[B32] Johnsen E, Jorgensen HA (2008). Effectiveness of second generation antipsychotics: a systematic review of randomized trials. BMC Psychiatry.

[B33] Correll CU, Leucht S, Kane JM (2004). Lower risk for tardive dyskinesia associated with second-generation antipsychotics: a systematic review of 1-year studies. Am J Psychiatry.

[B34] Kreyenbuhl JA, Valenstein M, McCarthy JF, Ganoczy D, Blow FC (2007). Long-term antipsychotic polypharmacy in the VA health system: patient characteristics and treatment patterns. Psychiatr Serv.

[B35] Ray WA, Chung CP, Murray KT, Hall K, Stein CM (2009). Atypical antipsychotic drugs and the risk of sudden cardiac death. N Engl J Med.

[B36] Tauscher J, Kapur S (2001). Choosing the right dose of antipsychotics in schizophrenia: lessons from neuroimaging studies. CNS Drugs.

[B37] Rijcken CA, Monster TB, Brouwers JR, de Jong-van den Berg LT (2003). Chlorpromazine equivalents versus defined daily doses: how to compare antipsychotic drug doses?. J Clin Psychopharmacol.

[B38] Hamann J, Langer B, Leucht S, Busch R, Kissling W (2004). Medical decision making in antipsychotic drug choice for schizophrenia. Am J Psychiatry.

[B39] Weinmann S, Janssen B, Gaebel W (2005). Guideline adherence in medication management of psychotic disorders: an observational multisite hospital study. Acta Psychiatr Scand.

